# Effect of Deep Eutectic System (DES) on Oral Bioavailability of Celecoxib: In Silico, In Vitro, and In Vivo Study

**DOI:** 10.3390/pharmaceutics15092351

**Published:** 2023-09-20

**Authors:** Soumalya Chakraborty, Rohit Y. Sathe, Jaydeep H. Chormale, Ashish Dangi, Prasad V. Bharatam, Arvind K. Bansal

**Affiliations:** 1 Department of Pharmaceutics, National Institute of Pharmaceutical Education and Research (NIPER), Sector-67, S.A.S. Nagar 160062, Punjab, India; sc280562@gmail.com (S.C.); jaydeep1591@gmail.com (J.H.C.); 2 Department of Medicinal Chemistry, National Institute of Pharmaceutical Education and Research (NIPER), Sector-67, S.A.S. Nagar 160062, Punjab, India; rohitysathe@gmail.com (R.Y.S.); pvbharatam@niper.ac.in (P.V.B.); 3 Department of Pharmacology and Toxicology, National Institute of Pharmaceutical Education and Research (NIPER), Sector-67, S.A.S. Nagar 160062, Punjab, India; ashishdangi0@gmail.com

**Keywords:** deep eutectic system, poorly soluble drugs, celecoxib, solubility, dissolution, oral bioavailability, density functional theory (DFT)

## Abstract

Different deep eutectic systems (DES) of choline chloride (CC)–urea (UA) (1:2), CC–glycerol (GLY) (1:2), CC–malonic acid (MA) (1:1), and CC–ascorbic acid (AA) (2:1) were generated and characterized by polarized light microscope (PLM) and Fourier transform infrared spectroscope (FTIR). The equilibrium solubility of celecoxib (CLX) in DES was compared to that in deionized water. The CC–MA (1:1) system provided ~10,000 times improvement in the solubility of CLX (13,114.75 µg/g) and was used for the generation of the CLX–DES system. The latter was characterized by PLM and FTIR to study the microstructure and intermolecular interaction between the CLX and CC–MA (1:1) DES. FTIR demonstrated the retention of the chemical structure of CLX. In vitro drug release studies in FaSSIF initially demonstrated high supersaturation, which decreased by ~2 fold after 2 h. Density functional theory (DFT)-based calculations provided a molecular-level understanding of enhanced solubility. Gibbs free energy calculations established the role of the strongest binding of CLX with CC and MA. A phase solubility study highlighted the role of hydrotropy-induced solubilization of the CLX–DES system. Animal pharmacokinetic studies established 2.76 times improvement in C_max_, 1.52 times reduction in t_max_, and 1.81 times improvement in AUC_0-∞_. The overall results demonstrated the potential of developing a DES-based supersaturating drug-delivery system for pharmaceutical loading of drugs having solubility and dissolution rate-limited oral bioavailability.

## 1. Introduction

Poor water solubility and the dissolution rate of molecules offer a major challenge in drug development [1]. Limited water solubility and a low dissolution rate can limit the oral bioavailability of a drug [2]. Hence, there is a strong need to develop new solubility-enabling formulation strategies.

Some recently published works [1,3,4,5,6] have highlighted the potential of deep eutectic system (DES) as a novel formulation to mitigate the delivery challenges associated with ‘difficult-to-deliver’, poorly soluble drugs. DESs are a class of eutectic mixtures comprised of two or more components. The strong hydrogen bonding interactions between DES constituents are responsible for significant lowering of the melting point in DES [7,8]. Until now, limited insights have been available for these systems, and they have often been mistaken for unstable cocrystals or solid solutions [1]. DESs are stable and have high solubilization capacity. They can improve the solubility, permeation, and absorption of drugs [9].

Limited studies have investigated the effect of DES on the oral bioavailability of poorly soluble drugs. Faggian et al. [10] demonstrated improved pharmacokinetics of rutin dissolved in proline–glutamic acid (2:1) DES compared to its aqueous suspension after oral administration in rats. There was no significant difference in the absorption rate (as evidenced by almost identical MRT value), but large differences in C_max_ and AUC were observed. Similar enhancement of the Cmax and AUC of berberine was demonstrated by Sut et al. [11], in which the oral pharmacokinetics of a berberine DES (proline–urea (2:1) DES and proline–malic acid–lactic acid–water (1:0.2:0.3:0.5) DES) formulation was compared with the aqueous suspension. A study by Chen et al. [12] suggested that oral administration of a salvianolic acid–DES formulation with a choline chloride–glycerol (1:2) DES resulted in enhanced Cmax and reduced Tmax compared to the corresponding aqueous suspension. Recently, Dangre et al. [13] showed that two-fold enhancement of the relative oral bioavailability of naringenin was achieved with a narigenin–DES formulation with choline chloride–glycerol (1:3) compared to aqueous suspension. However, more investigations are needed to completely understand the oral absorption-enabling potential of DES and the underlying mechanisms.

Celecoxib (CLX) is a non-steroidal anti-inflammatory drug (NSAID). Pharmacologically, CLX is a specific COX-2 inhibitor used to treat pain and inflammation [14]. A major downside of CLX is its low water solubility (3 mg L^−1^ at 37 °C) [15], which limits its oral bioavailability (22–40%) [16]. CLX is a BCS Class II drug (low solubility and high permeability) with a dose number of 80 [14].

In the current work, we investigated the DES for improving the oral bioavailability of CLX. Different DES were generated and screened to select the suitable one for developing a DES-based enabling system for CLX (CLX–DES system). The generated DES and CLX–DES system were characterized by microscopic and spectroscopic techniques. Performance evaluation of the CLX–DES system was carried out by in vitro release study and accelerated stability study. Density functional theory (DFT)–based calculations were performed to gain insights into the solubilization behavior of CLX in the selected DES. Different factors were investigated to elucidate the mechanism underlying the elevated supersaturation achieved with the CLX–DES system in in vitro release study under non-sink conditions. Finally, an in vivo oral pharmacokinetic study of CLX–DES system was performed in Sprague–Dawley rats.

## 2. Materials and Methods

### 2.1. Materials

CLX, chemically designated as 4-[5-(4-methylphenyl)-3-(trifluoromethyl)-1H-pyrazol-1-yl] benzenesulfonamide (Form III; assay value > 99%), was received as a gift from Zydus Healthcare Ltd. (Ahmadabad, India). Choline chloride (CC) (LobaChemie, Mumbai, India), urea (UA); (Sigma Aldrich, Bangalore, India), malonic acid (MA); (LobaChemie, India), glycerol (GLY); (Central Drug House, Delhi, India), and ascorbic acid (AA); (S.D. fine-chem Ltd., Vadodara, India) were of >98.0% purity. The solvents used were of high-performance liquid chromatography (HPLC) grade. All other chemicals used were of analytical grade.

### 2.2. Methods

#### 2.2.1. Preparation and Characterization of Prototype DES

DES of CC with other excipients (urea (UA), malonic acid (MA), glycerol (GLY), and ascorbic acid (AA)) were generated by the ‘heating method’, as reported elsewhere [17,18]. The selected excipient combinations were weighed (Model ME 204, Mettler Toledo India, Mumbai, India) and geometrically diluted at specific molar ratios (Table 1). The resulting mixture was sealed in a screw-capped glass vial and heated to a specified temperature (Table 1) in a magnetic stirrer coupled with a hot plate (IKA C-MAG HS7, IKA-Works GmBH&Co., Staufenim Breisgau, Germany) at a constant stirring rate until a homogeneous liquid solution was obtained. The generated systems were kept in a vacuum oven (Narang Scientific works, New Delhi, India) at 60 °C for 24 h for removal of residual moisture. The systems were kept in screw-capped glass vials and stored in desiccators in the presence of phosphorus pentoxide (offering 0% relative humidity) until further processing. The generated systems were characterized by PLM and FTIR. The excipient combinations, along with important material attributes and process parameters, are shown in Table 1.

The absence of solid particles in the generated DES was confirmed by PLM using a Leica DMLP polarized microscope (Leica Microsystem WetzlarGmbH, Wetzlar, Germany). After placing a few drops of DES underneath a cover slide, images were captured using a JVS color video camera at 200× magnification and were analyzed using Linksys software (Version 32).

For further understanding of the molecular-level interaction in the DES microstructure, FTIR spectroscopy of the pure components and DES was conducted. For each spectrum, 20 scans were measured between 4000 to 450 cm^−^^1^ with 2 cm^−^^1^ of spectral resolution on a standard FTIR spectrometer (PerkinElmer Inc., Waltham, MA, USA). The results were analyzed using Spectrum software (version 3.02.01).

#### 2.2.2. Equilibrium Solubility Determination of CLX in DESs

The equilibrium solubility of the drug was measured in DES and deionized water by the shake flask method [19] using shaker bath (Model SW23, JULABO GmbH, Seelbach, Germany). Briefly, CLX in excess quantity was added to 15-mL screw-capped vials containing 10 mL of medium (DES or deionized water). After vortexing for 5 min, the vials were kept in a shaker bath at 100 shakes/min at 25 °C to reach equilibrium conditions. At different time points (24 h, 48 h, and 72 h) 1 mL of the samples was withdrawn from each vial and centrifuged at 12,000 rpm for 10 min to ensure the sedimentation of undissolved drug. The supernatants were collected and were filtered using a 0.45-µm PTFE membrane syringe filter. The filtrate was diluted with methanol and analyzed by a validated HPLC method [15].

#### 2.2.3. Preparation and Characterization of Prototype CLX–DES System

The final CLX–DES system containing 8 mg CLX/g DES was prepared by dissolving CLX in DES at a 900-rpm stirring rate at an ambient temperature with a magnetic stirrer (IKA C-MAG HS7, Ika-Works GmBH&Co., Staufenim Breisgau, Germany). A transparent, clear, and viscous solution was generated and stored in a desiccator. The prepared CLX–DES formulation was further characterized by PLM and FTIR using the method described in Section 2.2.1.

#### 2.2.4. Accelerated Stability Study

An accelerated stability study was conducted on the CLX–DES system for 1 month in the stability conditions specified by the International Council for Harmonisation (ICH) of Technical Requirements for Pharmaceuticals for Human Use. The physical and chemical stability of CLX–DES formulation was assessed by placing the samples (in screw-capped glass vials) in a stability chamber at 40 °C/75% RH for 30 days. Samples were removed from the chamber after 15 and 30 days. Before testing, the samples were equilibrated to room temperature for a minimum of 24 hrs. The physical stability (lack of crystallinity) was assessed by PLM. At each time point, the recovered amount of the CLX–DES system was estimated by a validated HPLC method [15].

#### 2.2.5. In Vitro Drug Release Study

The in vitro drug release from the CLX–DES system was studied in fasted state simulated intestinal fluid (FaSSIF) using a USP dissolution apparatus type II (Model TDT 08L, Electrolab, Mumbai, India) at 37 °C and a 75-rpm stirring speed. The CLX–DES system (equivalent to 100-mg dry weight of CLX) was added in to 500 mL of pre-heated FaSSIF. Aliquots of media were withdrawn at 5, 15, 20, 30, 45, 60, 90, and 120 min and were immediately replaced with pre-heated FaSSIF. Dissolved drug concentrations at different time points were determined by a validated HPLC method [15], and the amount of dissolved drug was calculated considering the volume of replaced dissolution media. The same experiment was performed for crystalline CLX (100 mg). All in vitro drug release experiments were performed in triplicate.

#### 2.2.6. Solid State Characterization of Precipitate

After 120 min of drug release from the CLX–DES system, the precipitates were collected by passing the media through a 0.22-µm filter membrane. The collected precipitate was dried overnight in a vacuum oven (Narang Scientific Works, New Delhi, India) at 60 °C. The precipitate was further analyzed by PXRD (Rigaku Instruments, Akishima, Japan) and PLM using a Leica DMLP polarized microscope (Leica Microsystem Wetzlar GmbH, Germany).

#### 2.2.7. Phase Solubility Determination of CLX in Mixtures of DES and FaSSIF at Varying Proportions

The apparent solubility CLX was measured in neat FaSSIF, neat DES, and mixtures of DES with FaSSIF at varying weight proportions (20%, 40%, 60%, and 80% (*w*/*w*) DES) using a shaker bath (Model SW23, JULABO GmbH, Germany) by following the procedure mentioned elsewhere [20]. Briefly, 50 mg of CLX was added to 5-mL screw-capped vials containing 2 g of each media. After vortexing for 5 min, the vials were kept in a shaker bath at 100 shakes/min rate in 37 °C to reach equilibrium conditions. At different time points (24 h, 48 h, and 72 h) 1 mL of samples was withdrawn from each vial and centrifuged at 12,000 rpm for 10 min to ensure the sedimentation of undissolved drug. The supernatants were collected and filtered using a 0.45-µm PTFE membrane syringe filter. The filtrate was diluted with methanol and was analyzed by a validated HPLC method [15].

#### 2.2.8. Quantum Chemical (In Silico) Study

DFT calculations were performed to investigate the reason for the enhanced solubility of CLX in the CC–MA (1:1) DES. Interactions of components in CLX–DES system were described by considering homo- and hetero-molecular pairs. The thermodynamic characteristics of CLX–DES system were based on the Gibbs free energy of the X + Y = XY reaction type, where X and Y denote the considered species. The geometry optimization and the electrostatic potential (ESP) maps in 3D generation were performed with the hybrid-functional B3LYP, along with the 6-311+G(d) split-valance basis set, employing the polarization and diffuse functions for the heavy atoms in the systems. All calculations were performed with the Gaussian 16 program package.

#### 2.2.9. In Vivo Pharmacokinetic Study

In vivo oral pharmacokinetic studies of the CLX–DES system were performed in female Sprague–Dawley rats (weighed 200 ± 30 g) according to the protocol (IAEC/21/89) approved by Institutional Animal Ethics Committee, NIPER, S.A.S. Nagar, India. All the animals were acclimatized for 1 week by exposing them to 12 h of periodic light and dark conditions at 25 °C and 60% RH. Before the study, the animals were divided into two groups consisting of five animals each (group 1: CLX–DES system; and group 2: pure CLX) and were fasted overnight with free access to water. A dose of 5 mg of CLX/kg of the rat body mass was administered to both the groups via oral gavage. Blood samples (150–200 μL) were collected from the tail vein at 0.5, 1, 2, 4, 8, 12, 24, and 48 h. Plasma from the blood samples was separated by centrifugation at 7000 rpm for 7 min at 4 °C and stored at −20 °C until analysis. Analysis of plasma samples was performed using a validated bioanalytical method [21]. Pharmacokinetic analysis was performed from the mean plasma concentration–time profiles using the PK Solver (version 2.0) add-on program in Microsoft Excel 2019 software (Microsoft Corporation, Redmond, WA, USA).

## 3. Results and Discussion

### 3.1. Characterization of DES

The generated DESs were characterized by PLM and FTIR. PLM works as an indispensable tool for the characterization of DES to detect the existence of solid components. In cases of unfavorable interactions between the components, the presence of solid crystalline particles can be identified from the presence of birefringence, although the system may be a transparent liquid to the naked eye [22]. Lack of birefringence in the PLM images of DES (Figure S1) confirmed the liquid microstructure of DES in the generated systems.

FTIR spectroscopy helped to describe the interactions among the DES components. Interaction can be evident from the widening or overlapping of the bands consisting of hydroxyl and amino groups, the shift of the carbonyl band of carboxyl moiety, or the absence of the characteristic group peak of pure substances [23]. FTIR spectra of all the pure components and their corresponding DESs are captured in Figures S2 and S3.

The FTIR spectra of CC demonstrated the vibrational band at 3358 cm^−1^ due to the −OH or −NH_2_ group stretching; at 3025 cm^−1^ and 2961 cm^−1^ due to the alkyl group; and at 1479 cm^−1^ due to the CH_2_ group bending [24].

The FTIR spectra of MA exhibited a broad peak at 1707 cm^−1^ due to the carbonyl stretching; and strong, broad, overlapping peaks at 3000 cm^−1^ due to hydrogen-bonded dimer rings in carboxylic acids. In the FTIR spectra of CC–MA (1:1) DES, a shift of the carbonyl stretching band of MA to a higher frequency (1731 cm^−1^) and a reduction in intensity were observed, indicating the involvement of the carbonyl group of malonic acid in DES formation. The broad, overlapping peaks at the OH stretching region of MA were also present in the FTIR spectra of the CC–MA (1:1) DES, indicating the existence of multiple indistinguishable hydrogen bonds in this region [25].

In agreement with previous studies [26,27], the FTIR spectra of the CC–GLY (1:2) DES demonstrated various characteristic peaks of N–C–C bending (956 cm^−1^), C-H stretching (2935 cm^−1^), hydrogen bond liberation overtones (2128 cm^−1^) and N–H and O–H stretching (3364 cm^−1^). It is interesting to observe that the GLY and CC–GLY (1:2) DES showed similar FTIR spectra due to the same IR active functional groups.

In the FTIR spectra of the CC–UA (1:2) DES, an increase in the carbonyl stretching frequency of UA (1619 cm^−1^) to 1666 cm^−1^ and 1621 cm^−1^ indicated the formation of the DES [17].

In the FTIR spectra of AA, the carbonyl stretching band at 1739 cm^−1^ showed a significant, positive shift (1760 cm^−1^) after formation of the CC–AA (2:1) DES. Additionally, in the DES, the C–H stretching vibration of CC at 3025 cm^−1^ disappeared, indicating the formation of hydrogen bonding between the components [28].

### 3.2. Equilibrium Solubility of CLX in DES

CC–UA (1:2), CC–GLY (1:2), CC–MA (1:1), and CC–AA (2:1) DES were used to screen the solubility of CLX at 25 °C. Deionized water (pH~5) was used as the reference solvent. The solubility of CLX in deionized water at 25 °C was found to be very low (1.21 µg/g). The solubility of CLX in DES is shown in Table 2.

CLX demonstrated maximum solubility enhancement in the CC–MA (1:1) DES. MA is an aliphatic dicarboxylic acid with an encouraging safety profile. It has also been extensively used as a coformer for the generation of cocrystals of many drugs [29,30,31,32,33]. Previous studies have suggested the use of the ‘solubility’ of a drug as a basis for screening DES for fabricating DES-based drug delivery systems [10,11]. Based on maximum solubility enhancement and an encouraging safety profile, the CC–MA (1:1) DES was selected for generation of CLX–DES formulation for further development.

### 3.3. Characterization of CLX–DES System

PLM images (Figure S4) of the CLX–DES system did not show any birefringence, indicating the absence of crystalline CLX in the system.

**Figure 1:**
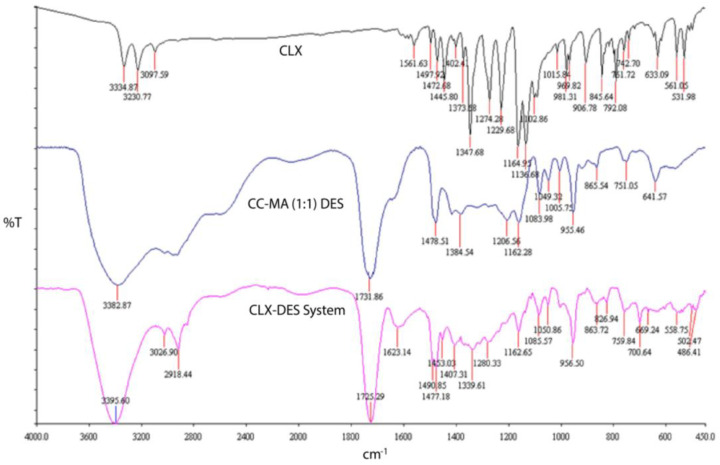
FTIR spectra of pure CLX, the CC–MA (1:1) DES, and the CLX–DES system.

To understand the intermolecular interactions between CLX and the CC–MA (1:1) DES components, the infrared spectra of pure CLX, as well as the CLX–DES system, were performed. FTIR spectra of pure CLX and the CLX–DES system are demonstrated in Figure 1.

FTIR spectra of pure CLX demonstrated bands between 3000 to 3350 cm^−1^ (NH_2_ group stretching frequency), a band at 1561 cm^−1^ (N-H group stretching), and a band at 1100 cm^−1^ to 1350 cm^−1^ (sulfonamide group stretching) [34].

Interestingly, the FTIR spectrum of the CLX–DES system was a combination of the pure CLX and CC–MA (1:1) DES spectra. All the previous bands were clearly observed in the spectrum of the CLX–DES system, indicating the intact structural integrity of CLX in the CLX–DES system. The hydroxyl stretching band of CC at 3398 cm^−1^ of the pure DES spectrum became sharpened with an increase in the intensity of the CLX–DES (2:1) spectrum. The frequency of the carbonyl stretching band of MA in the pure CC–MA (1:1) DES increased in the CLX–DES system (1725 cm^−1^), pointing toward the hydrogen bond formation. The retaining of original IR bands indicates the structural integrity of the chemical structure of CLX in the CLX–DES system.

### 3.4. Accelerated Stability Study

Results of accelerated stability studies are shown in Table S1. PLM images of periodically collected samples demonstrated the absence of birefringence, indicating the preservation of the liquid microstructure of the system in the aforementioned conditions. Assay values of CLX were within acceptable limits, indicating their uninterrupted chemical stability in the system over time.

### 3.5. In Vitro Drug Release Study

In vitro drug release study was performed for both pure crystalline CLX and the CLX–DES system. CLX exhibits ~46 µg/mL solubility in FaSSIF media at 37 °C. Thus, the selected experimental media for in vitro release study provided a non-sink environment, facilitating exploration of the supersaturation potential of the CLX–DES system.

**Figure 2:**
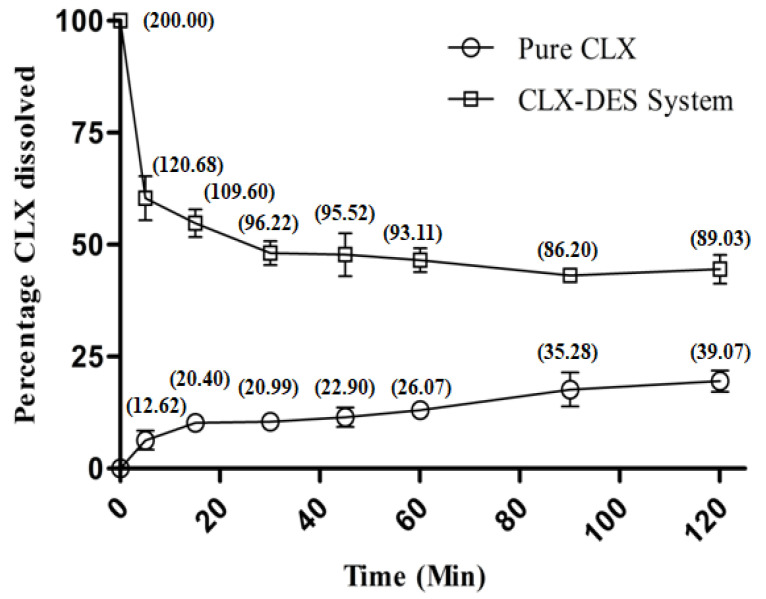
Percentage CLX dissolved with time (bracketed values indicate the concentration in µg/mL).

The CLX–DES system containing 8 mg CLX/g DES was introduced into FaSSIF. The system was added to achieve a target concentration of 200 µg/mL of CLX in 500 mL of medium. Initially, a clear, supersaturated solution was observed that was followed by rapid precipitation (with visible precipitates) at the subsequent time points (Figure 2).

After 2 h, the CLX–DES system demonstrated an ~89-µg/mL concentration, which was ~2 fold higher than CLX. Thus, the CLX–DES system demonstrated the ability to achieve supersaturation for a biopharmaceutically relevant time frame.

### 3.6. Mechanistic Investigation of Enhanced Solubility of CLX in the CC–MA (1:1) DES

The experimentally observed elevated solubility of CLX in the CC–MA (1:1) DES was investigated at the molecular level by studying detailed mechanisms of inter- and intra-molecular affinity. Thermodynamic characteristics can be quantified from the values of the Gibbs free energies of reactions for homo-or hetero-molecular pair formation among solution components, which lead to the formation of molecular complexes. Therefore, DFT-based first-principles calculations were performed to obtain the structure of systems comprising CLX, CC, and MA in experimental proportions. For all optimized geometries (Figure S5), all vibrational frequencies were found positive (i.e., imaginary frequencies are absent), signifying the ground state of these structures. The atom legends used in these geometries are: hydrogen: white; carbon: gray; nitrogen: blue; oxygen: red; fluorine: aqua-blue; sulfur: yellow; and chlorine: lime green. These geometries are presented along with the ESP maps of a respective structure. In the ESP maps, the red color denotes the region with high electron density, the yellow color denotes the region with moderately high electron density, the green color denotes a charge-neutral region, the sky-blue color denotes a moderately electron-deficient region, and blue color denotes the region with low electron.

**Figure 3:**
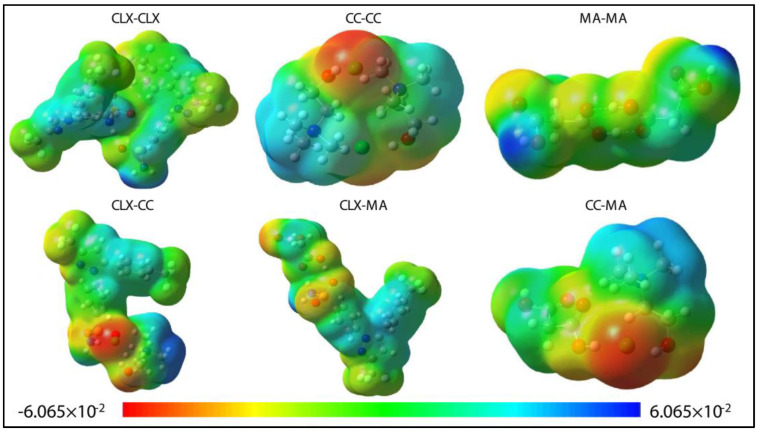
ESP maps of the stable homo– and hetero–molecular dimers of the CLX–DES formulation. The unit of the color scale is e/Å^3^.

The pair formation reaction can be indicated as: X + Y = XY, where X, Y= {CLX, CC, MA}, and the values of Gibbs free energy of pair formation express the strength of affinity of components toward each other. Considering three solution components, three homo-molecular (CLX–CLX, CC–CC, MA–MA) and three hetero-molecular (CLX–CC, CLX–MA, CC–MA) pairs were identified. All combinations were considered for identification of the driving forces of solubility advantage. Electrostatic surface potential (ESP) maps of these pairs are presented in Figure 3.

Contributions of all the structures to the overall stabilization of the system can be inferred from the computed values of Gibbs free energies of reactions. Maximum stabilization was observed for the CLX–CC and CLX–MA heteromolecular complexes, indicating the role of the strongest binding of CC and MA with CLX in the CLX–DES system microstructure in maintaining the bound form of CLX in the system and hence increasing its solubility in the CC–MA (1:1) DES. A similar tendency of curcumin in forming energetically favorable heteromolecular complexes with both choline chloride and glycerol was observed in the study of Jelinsky et al. [4], and it was identified as the main contributor of enhanced solubility of curcumin in the choline chloride–glycerol (1:1) DES.

Overall, in the present study, the relative stabilization observed in the CLX–DES system followed the trend of CLX–CC (−67.52 kcal/mol) ≈ CLX–MA (−63.56 kcal/mol) > CC–CC (−18.08 kcal/mol) > CLX–CLX (−14.87 kcal/mol) > MA–MA (−10.56 kcal/mol) > CC–MA (−9.70 kcal/mol). CLX homomolecular complexes did not contribute to the solubility increase due to much lower stability compared to hetero-molecular pairs.

### 3.7. Mechanistic Investigation of Improved Drug Release of the CLX–DES Formulation

The involvement of hydrotropy and co-solvency as principal mechanisms in DES and ionic liquids (IL)-induced solubilization was first explored by the work of Coutinho and coworkers [35].

Hydrotropes are compounds with the capacity to improve the solubility of hydrophobic solutes. Although numerous research papers have depicted the ability of hydrotropes to enhance the solubilization of hydrophobic drugs, the underlying mechanisms are not yet clearly understood [36]. The main proposed mechanism are: (i) formation of a solute–hydrotrope complex; (ii) hydrotrope-induced alteration of solvent structures (formation of chaotropes and chasmotropes); and (iii) the co-aggregation of the solutes with hydrotropes. Hydrotropy is characterized by a sigmoidal solubilization profile of the solute with increasing hydrotrope concentrations [20,37].

Co-solvents are water-soluble compounds and can increase the solubility of hydrophobic compounds. This solubilization process is induced by the mixed solvent (water+co-solvent) and has a solvation ability that is intermediate to that of the pure water and the co-solvent. Unlike hydrotropy, the solubilization achieved by the co-solvent is a linear or monotonic function of its concentration [35].

**Figure 4:**
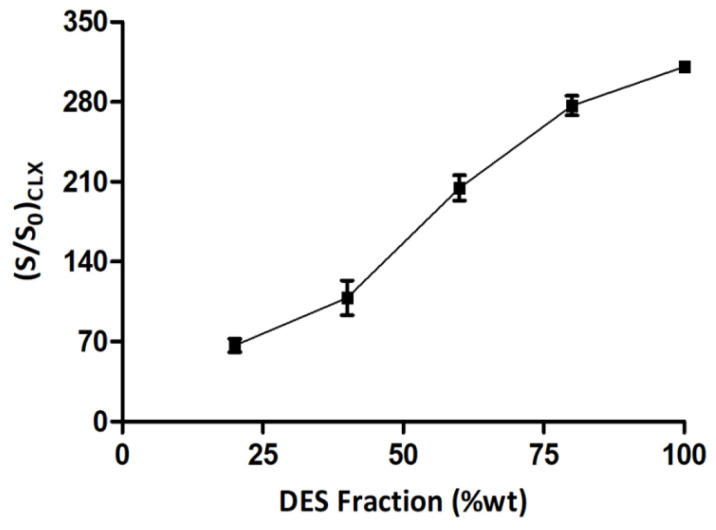
Influence of DES concentration on CLX solubility.

In the present study, the phase solubility of CLX was estimated in FaSSIF media at 37 °C with an increasing weight fraction of the CC–MA (2:1) DES to elucidate the mechanism of solubilization. The influence of DES concentration on CLX solubility is illustrated in Figure 4.

The sigmoidal solubility profile of CLX in a gradually increasing weight fraction of the CC–MA (1:1) DES in FaSSIF is indicative of the involvement of hydrotropic interactions in the solubilization process.

However, the exact mechanism of hydrotropy-induced solubilization of the CLX–DES system is difficult to decipher due to the possible involvement of multiple mechanisms. Additionally, it is important to mention that the bile salt (sodium taurocholate) and phospholipid (egg lecithin) present in FaSSIF may participate in the overall interaction during solubilization. This aspect requires further investigation to elucidate the complete mechanism behind DES-induced hydrotropic solubilization, especially in biorelevant media.

An attempt was made to capture SEM images of hydrotropic aggregates. However, this approach did not work due to interference of the vesicle structures present in blank FaSSIF. The deconvolution of possible aggregates from the vesicles present in blank FaSSIF was not possible (Figure S6).

To elucidate the occurrence of the high-energy solid form in the precipitate during the drug release study of the CLX–DES system, precipitates were collected after 2 h and subsequently were analyzed with PLM and PXRD. The high-energy solid form could contribute to solubility advantages.

**Figure 5:**
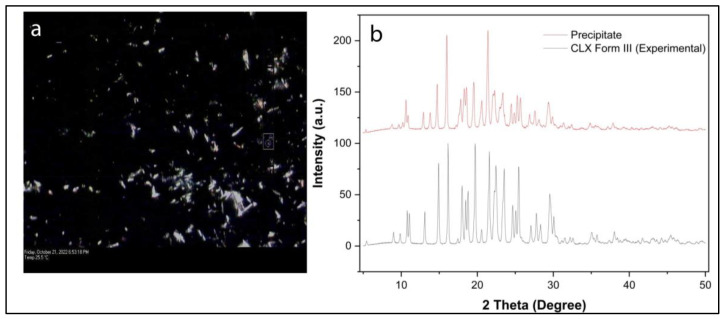
(**a**) PLM image of precipitate (bright birefringence eliminates the chance of amorphous nature of precipitate); (**b**) PXRD overlay of precipitate with powder CLX and predicted pattern of form III (stable at ambient condition).

PLM images (Figure 5a) revealed the needle-shaped crystalline nature of the precipitates with bright birefringence, eliminating the possibility of the amorphous form of CLX in the precipitate. A PXRD diffractogram (Figure 5b) revealed the presence of a stable polymorphic form (form III) of CLX with characteristics peaks at 5.362, 9.687, 10.622, 14.729, 17.816, and 21.42 [38]. It indicates that the formation of the metastable polymorphic form of CLX did not take place during precipitation.

During in vitro release study of the CLX–DES system (Figure 2), it was observed that CLX was completely dissolved at the beginning (t = 0), imparting maximum supersaturation. At subsequent time points, decreases in the initial supersaturation occurred, but the dissolved drug concentration was still significantly higher than the thermodynamic solubility of CLX. The CLX–DES system was able to maintain the supersaturation of CLX for up to 2 h. The fall in the initial supersaturation can be explained from the water-mediated disruption of the hydrogen-bonding network of the DES. The effect of water on the microstructure of DES has been widely studied [39,40,41,42,43], and it was identified that water at high concentrations, owing to its hydrogen bond donor/acceptor property (due to the hydrogen atoms and lone pair of electrons on oxygen atoms, respectively), competes with the DES components to form new hydrogen bonds, leading to a perturbation/disruption in the DES microstructure. However, this effect is not immediate and initiates on crossing a threshold value, which is based on the nature of the DES components and temperature. Maintenance of supersaturation can be explained based on competing mechanisms of precipitation and hydrotropy. The sigmoidal shape of the phase solubility plot suggests the participation of a hydrotropy mechanism in CLX solubilization. Thus, competition between two simultaneously occurring counter-intuiting driving forces, namely precipitation and hydrotropic solubilization, were responsible for the overall solubilization behavior of CLX from the CLX–DES system in FaSSIF.

Thus, prominent evidence for the role of hydrotropy in the drug release performance of CLX–DES was established. However, as reported earlier [35], involvement of the hydrotropy mechanism in DES or IL-mediated solubilization is not common for all systems and is highly dependent on the nature of the solute and the components of DES or IL. Thus, aforementioned mechanistic explanations are specific to the present study and cannot be generalized to other DES.

### 3.8. In Vivo Pharmacokinetic Study

**Figure 6:**
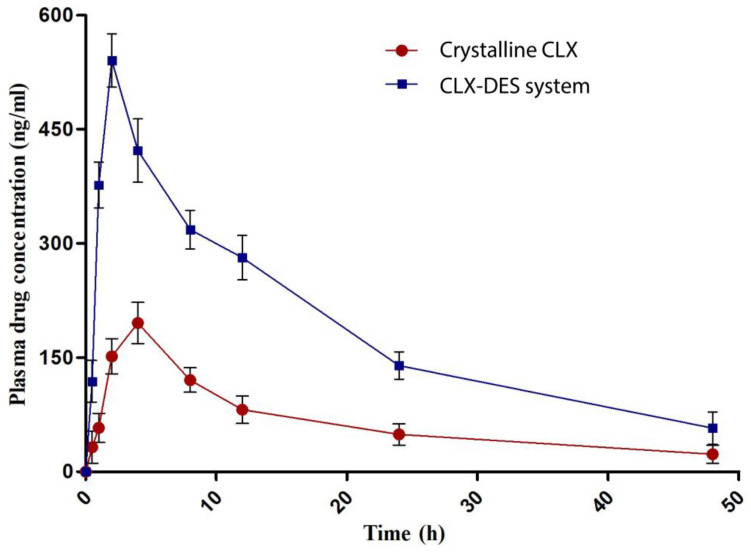
Plasma concentration–time profile of crystalline CLX and the CLX–DES system.

In in vivo pharmacokinetic studies, CLX–DES formulation demonstrated greater plasma concentration-time profiles than crystalline CLX (Figure 6).

The maximum plasma concentration (Cmax) achieved from the CLX–DES system was 540.41 ng/mL at 2.7 h. whereas pure crystalline CLX exhibited a Cmax of 195.39 ng/mL at 4.1 h. The values of Cmax and Tmax and the area under the plasma concentration time curve (AUC) are summarized in Table 3.

AUC is a measure of the extent of total systemic exposure to the drug after administration. It is calculated by integrating the plasma concentration values over time rather than examining individual concentrations; thus, a more accurate estimate of the overall exposure to the drug is obtained [44]. In the present study, the CLX–DES system demonstrated 1.81-fold enhancement in AUC compared to the crystalline CLX, indicating improvement of the ability of CLX to reach the systemic circulation after oral administration. The CLX–DES system also showed a 1.5-fold reduction in T_max_ and a 2.76-fold enhancement in C_max_.

This enhancement in the AUC and C_max_ can be attributed to the enhanced apparent solubility and supersaturation of CLX from the CLX–DES system. In vitro release study in FaSSIF demonstrated that supersaturation of CLX was maintained for up to 2 h. The presence of CLX in the dissolved state in the gastrointestinal tract for prolonged periods maintained the concentration gradient and increased the systemic absorption of CLX, leading to increases in AUC and C_max_. It was also observed in in vitro release study that the initial (t = 0 h) maximum CLX supersaturation from the CLX–DES system decreased at the subsequent time points due to precipitation. This finding can be correlated with the reduction in T_max_ observed in the pharmacokinetic study. At the initial time points, the degree of supersaturation of CLX was very high, leading to rapid absorption and the attaining of C_max_ within a considerably short period of time (T_max_).

## 4. Insights on DES-Based Formulation Development

DES belong to the supersaturable drug delivery systems, which impart biopharmaceutical advantages by maintaining the supersaturable state of drugs in the gastrointestinal tract, leading to improved systemic absorption. Unlike other supersaturable drug-delivery systems (amorphous solid dispersion, cocrystals), DES are thermodynamically stable without having a propensity to revert to the original state [45]. The preparation methods and commonly used excipients are simple, cost-effective, and scalable. Additionally, owing to their ‘tailor-made’ nature, the properties of DES can be easily tuned per the need of the drug molecule by changing the qualitative composition and molar ratio of the components [7].

The major drawback of the DES is its phase separation after dilution with the gastrointestinal fluid, causing precipitation of the drug. As the DES is a liquid supersaturable drug-delivery system, it produces a highly supersaturated solution after oral administration and gastrointestinal dilution. However, this initial supersaturation is lost at the subsequent stage due to the destructive impact of water on the DES microstructure [39]. The previous research work [46] on a solid supersaturable self-emulsifying drug-delivery system (SS-SEDDS) of CLX, published from our laboratory, demonstrated that, at same dose level (5 mg/kg) and with the same animal species (female Sprague–Dawley rat) as the current study, the SS-SEDDS of CLX showed 2.31-fold and 4.82-fold improvement in the C_max_ and AUC of CLX, respectively, compared to crystalline CLX. The improvement in the AUC with the SS–SEDDS was higher than with the CLX–DES system in the current study, which can be attributed to the absence of any precipitation inhibitor in the CLX–DES system. The SS-SEDDS contained solubilizer (tween 20) and precipitation inhibitor (soluplus), that helped in higher solubilization and prolonged supersaturation, leading to higher AUC. Thus, suitable precipitation inhibitors (mostly polymers) should be screened for the development of prototype DES-based formulations to harness maximum biopharmaceutical advantages. DES-filled soft gelatin capsules or liquid–solid compacts are some of the possible formulation approaches. Screening of the capsule shell should be carefully performed to retain the solubility benefit of the DES. Another important concern is the toxicity of the DES. To minimize the risk of toxicity, judicious selection of excipients and their concentrations in DES is required for formulation development.

Optimization of the qualitative and quantitative composition of DES-based formulations has the potential of deciphering comparable biopharmaceutical advantages with other enabling technologies. Palmelund et al. compared nanocrystalline, amorphous, and DES-based formulations (containing HPMC) of aprepitant for biopharmaceutical advantages, and they concluded that the DES formulation (34 ± 4%) showed higher oral bioavailability than amorphous aprepitant (20 ± 4%) and was on par with the oral bioavailability obtained from the nanocrystalline formulation (36 ± 2%) [9].

## 5. Conclusions

This study investigated the impact of DES on the in vitro and in vivo performance of CLX. The equilibrium solubility of CLX in the CC–MA (1:1) DES at room temperature was 10,838 times higher than its solubility in deionized water. DFT-based first-principles calculations indicated that the direct intermolecular contacts that led to the formation of hetero-molecular pairs of CLX with CC and MA are the most stable among all possible homo- and hetero-molecular pairs that can be found in the CLX–DES system and are responsible for the elevated solubility of CLX in the CC–MA (1:1) DES. The in vitro drug release study of the CLX–DES system demonstrated that supersaturation of dissolved CLX in FaSSIF was achieved in the presence of the DES. There was a decrease in initial supersaturation; however, significantly higher solubility was still observed. This outcome could indicate the simultaneously competing processes of precipitation and hydrotropic solubilization. The CLX–DES system exhibited improved pharmacokinetic performance after oral administration. These findings have implications for the development of DES-based enabling formulations of solubility and/or dissolution rate-limited drugs.

## Figures and Tables

**Table 1:** Excipient combinations along with important material attributes and process parameters.

Excipient Combination		Molar Ratio	Temperature (°C)	Mixing Rate (rpm)
Hydrogen Bond Acceptor (HBA)	Hydrogen Bond Donor (HBD)			
Choline chloride (CC) 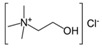 Molar Mass= 139.62 g/mole	Urea (UA) 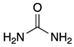 Molar Mass= 60.06 g/mole	1:2	80	500
	Glycerol (GLY) 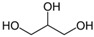 Molar Mass= 92.09 g/mole	1:2	80	500
	Malonic acid (MA) 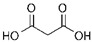 Molar Mass= 104.06 g/mole	1:1	60	500
	Ascorbic acid (AA) 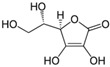 Molar Mass= 176.12 g/mole	2:1	80	500

**Table 2:** Equilibrium solubility of CLX in DESs at 25 °C.

DES	Equilibrium Solubility; *S_DES_*(µg/g) (*S_water_* = 1.21 ± 0.02 µg/g)	Enhancement Factor (*S = S_DES_/S_Water_*)
CC–UA (1:2)	1562.50 ± 7.16	1291
CC–GLY (1:2)	5383.90 ± 5.95	4449
CC–MA (1:1)	13,114.8 ± 6.03	10,838
CC–AA (2:1)	2364.73 ± 2.48	1954

**Table 3:** Pharmacokinetic parameters evaluated for crystalline CLX and CLX–DES system.

Pharmacokinetic Parameter	Crystalline CLX	CLX–DES System	Order of Change in CLX–DES System
C_max_ (ng/mL)	195.39	540.41	2.76
T_max_ (h)	4.1	2.7	1.52
AUC_0–∞_ (ng/mL.h)	5708.04	10,354.8	1.81

## Data Availability

The authors confirm that the data supporting the findings of this study are available within the article.

## Supplementary Materials

The following supporting information can be downloaded at: https://www.mdpi.com/article/10.3390/pharmaceutics15092351/s1, Figure S1: Optical (a–d) and PLM (e–h) images of DES of (a,e) CC–UA (1:2), (b,f) CC–GLY (1:2), (c,g) CC–MA (1:1), and (d,h) CC–AA (2:1); Figure S2: FTIR spectra of pure components; Figure S3: FTIR spectra of DES.; Figure S4:Optical (a) and polarized (b) microscopic images of pure CLX–DES system; Figure S5: Optimized geometries of all the possible homo- and hetero-molecular pairs of CLX, CC, and MA; Figure S6: SEM image of blank FaSSIF containing vesicular structures. Table S1: Percentage assay value and PLM images of the CLX–DES system after 15 and 30 days of storage at 40 °C/75% RH.
