# Supplementary material for: Effect of Deep Eutectic System (DES) on Oral Bioavailability of Celecoxib: In Silico, In Vitro, and In Vivo Study
Source: Pharmaceutics. 2023 Sep 20;15(9):2351. doi: 10.3390/pharmaceutics15092351 (PMC10534828; doi:10.3390/pharmaceutics15092351)
Supplement: Supplementary file 1 [file pharmaceutics-15-02351-s001.zip › pharmaceutics-2575800-supplementary.pdf]

pharmaceutics

MDPI

Effect of Deep Eutectic System (DES) on Oral Bioavailability of Celecoxib: In Silico, In Vitro, and In Vivo Study

Soumalya Chakraborty [1], Rohit Y. Sathe [2,†], Jaydeep H. Chormale [1], Ashish Dangi [3], Prasad V. Bharatam [2] and Arvind K. Bansal [1,*]

[1] Department of Pharmaceutics, National Institute of Pharmaceutical Education and Research (NIPER), Sector-67, S.A.S. Nagar 160062, Punjab, India; sc280562@gmail.com (S.C.); jaydeep1591@gmail.com (J.H.C.)
[2] Department of Medicinal Chemistry, National Institute of Pharmaceutical Education and Research (NIPER), Sector-67, S.A.S. Nagar 160062, Punjab, India; rohitysathe@gmail.com (R.Y.S.); pvbharatam@niper.ac.in (P.V.B.)
[3] Department of Pharmacology and Toxicology, National Institute of Pharmaceutical Education and Research (NIPER), Sector-67, S.A.S. Nagar 160062, Punjab, India; ashishdangi0@gmail.com
* Correspondence: akbansal@niper.ac.in; Tel.: +91-172-2214682-2126; Fax: +91-172-2214692
† Current Address: DBT-ICT Centre for Energy Biosciences, Institute of Chemical Technology, Mumbai 400019, Maharashtra, India.

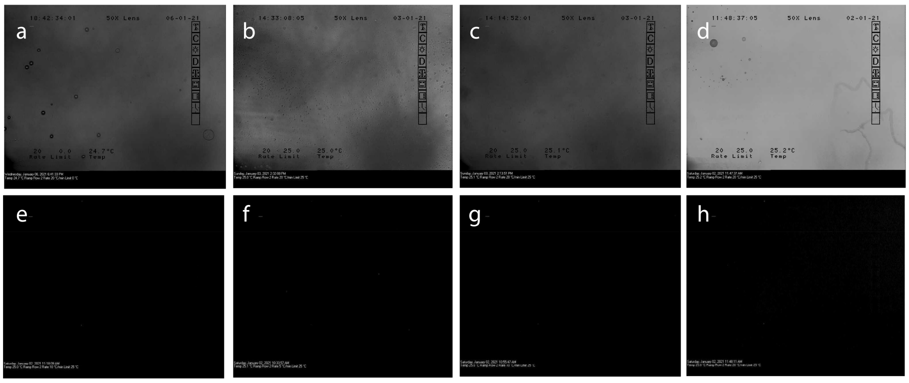

Figure S1. Optical (a–d) and PLM (e–h) images of DES of (a,e) CC–UA (1:2), (b,f) CC–GLY (1:2), (c,g) CC–MA (1:1), and (d,h) CC–AA (2:1).

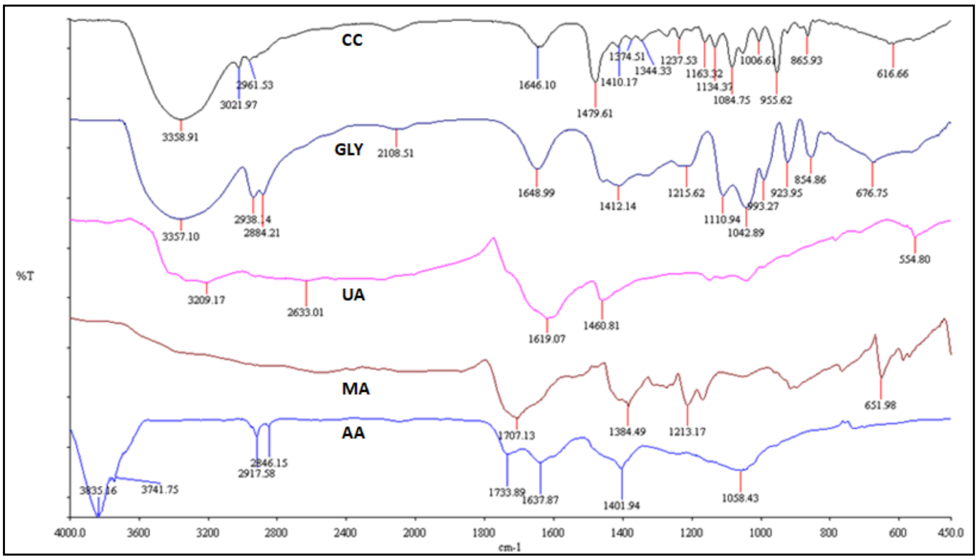

Figure S2. FTIR spectra of pure components.

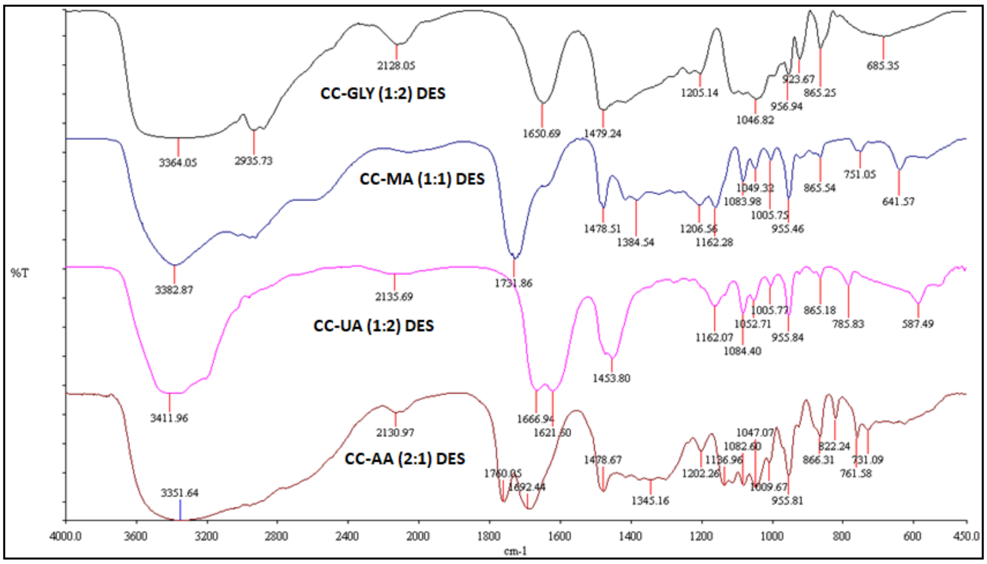

Figure S3. FTIR spectra of DESs.

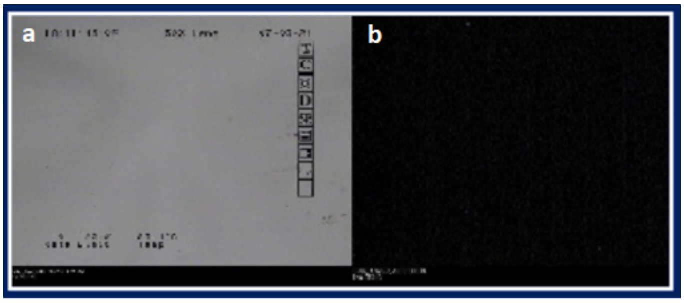

Figure S4. Optical (a) and polarized (b) microscopic images of pure CLX-DES system.

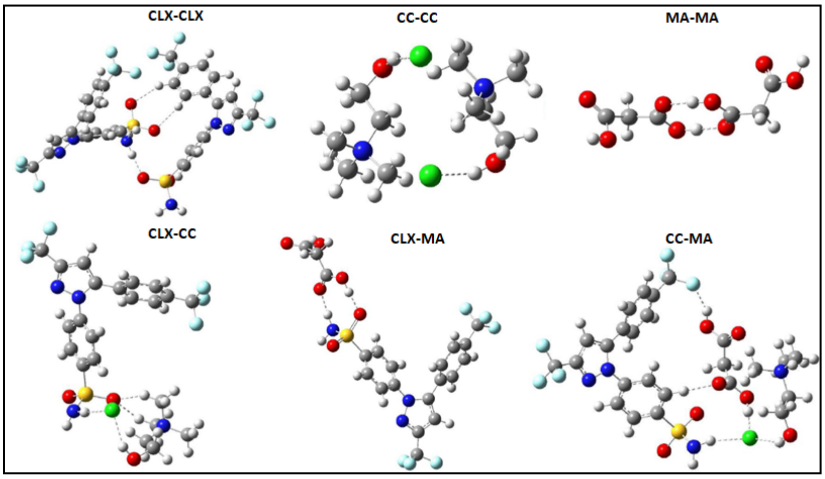

Figure S5. Optimized geometries of all the possible homo- and hetero-molecular pairs of CLX, CC, and MA.

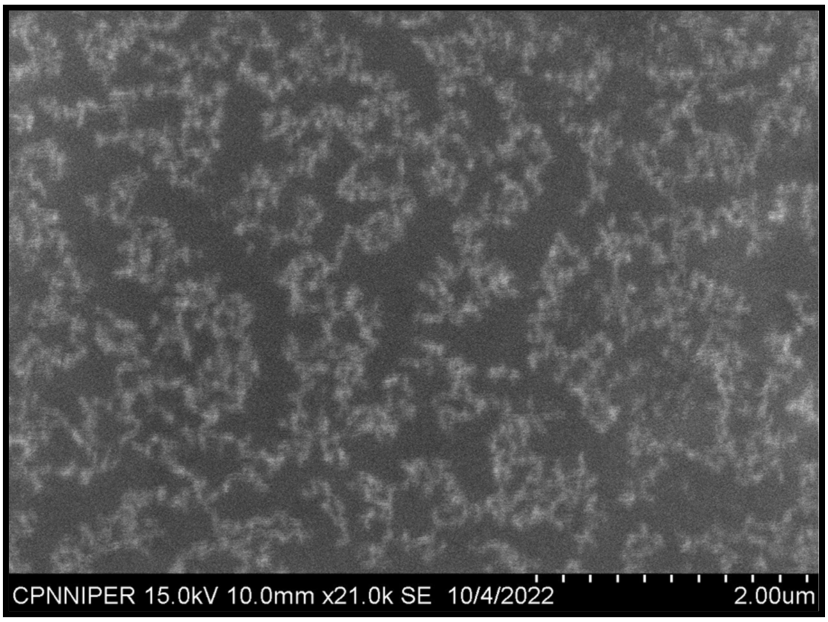

Figure S6. SEM image of blank FaSSIF containing vesicular structures.

Table S1. Percentage assay value and PLM images of CLX-DES system after 15 and 30 days storage at 40°C/75% RH.

Time (Days)	Assay (%)	PLM
0	100.01	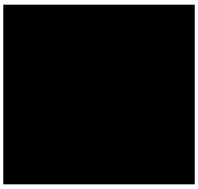
15	99.15	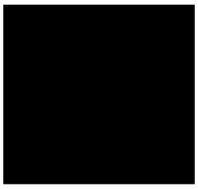
30	99.72	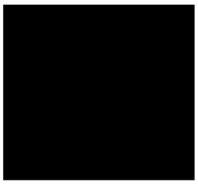
